# Transcriptome profiling of genes regulated by phosphate-solubilizing bacteria *Bacillus megaterium* P68 in potato (*Solanum tuberosum* L.)

**DOI:** 10.3389/fmicb.2023.1140752

**Published:** 2023-04-17

**Authors:** Lizhen Lin, Chengchen Li, Zongling Ren, Yuzhi Qin, Ruilong Wang, Jia Wang, Jianying Cai, Lanfeng Zhao, Xiaobo Li, Yanfei Cai, Xingyao Xiong

**Affiliations:** ^1^College of Natural Resources and Environment, South China Agricultural University, Guangzhou, China; ^2^Guangdong Provincial Key Laboratory of Crop Genetic Improvement, Crops Research Institute, Guangdong Academy of Agricultural Sciences, Guangzhou, China; ^3^Agricultural Genomics Institute at Shenzhen, Chinese Academy of Agricultural Sciences, Shenzhen, China; ^4^Engineering Research Center for Horticultural Crop Germplasm Creation and New Variety Breeding, Ministry of Education Changsha, Hunan Provincial Engineering Research Center for Potatoes, Southern Regional Collaborative Innovation Center for Grain and Oil Crops in China, Key Laboratory for Vegetable Biology of Hunan Province, College of Horticulture, Hunan Agricultural University, Changsha, China; ^5^Key Laboratory of Agro-Environment in the Tropics, Ministry of Agriculture, College of Natural Resources and Environment, South China Agricultural University, Guangzhou, China; ^6^Guangdong Institute Center of Wine and Spirits, Guangdong Institute of Food Inspection, Guangzhou, China

**Keywords:** *Bacillus megaterium*, transcriptome, potato, commercial tubers yield, phosphate solubilizing bacteria

## Abstract

The insoluble phosphorus in the soil is extremely difficult to be absorbed and used directly through the potato root system. Although many studies have reported that phosphorus-solubilizing bacteria (PSB) can promote plant growth and uptake of phosphorus, the molecular mechanism of phosphorus uptake and growth by PSB has not been investigated yet. In the present study, PSB were isolated from rhizosphere soil in soybean. The data of potato yield and quality revealed that the strain P68 was the most effective In the present study, PSB identification, potato field experiment, pot experiment and transcriptome profiling to explored the role of PSB on potato growth and related molecular mechanisms. The results showed that the P68 strain (P68) was identified as *Bacillus megaterium* by sequencing, with a P-solubilizing ability of 461.86 mg·L^−1^ after 7-day incubation in National Botanical Research Institute’s Phosphate (NBRIP) medium. Compared with the control group (CK), P68 significantly increased the yield of potato commercial tubers by 17.02% and P accumulation by 27.31% in the field. Similarly, pot trials showed that the application of P68 significantly increased the biomass, total phosphorus content of the potato plants, and available phosphorus of the soil up by 32.33, 37.50, and 29.15%, respectively. Furthermore, the transcriptome profiling results of the pot potato roots revealed that the total number of bases was about 6G, and Q30 (%) was 92.35–94.8%. Compared with the CK, there were a total of 784 differential genes (DEGs) regulated when treated with P68, which 439 genes were upregulated and 345 genes were downregulated. Interestingly, most of the DEGs were mainly related to cellular carbohydrate metabolic process, photosynthesis, and cellular carbohydrate biosynthesis process. According to the KEGG pathway analysis, a total of 46 categorical metabolic pathways in the Kyoto Encyclopedia of Genes and Genomes (KEGG) database were annotated to 101 DEGs found in potato roots. Compared with the CK, most of the DEGs were mainly enriched in glyoxylate and dicarboxylate metabolism (sot00630), nitrogen metabolism (sot00910), tryptophan metabolism (sot00380), and plant hormone signal transduction (sot04075), and these DEGs might be involved in the interactions between *Bacillus megaterium* P68 and potato growth. The qRT-PCR analysis of differentially expressed genes showed that inoculated treatments P68 significantly upregulated expression of the phosphate transport, nitrate transport, glutamine synthesis, and abscisic acid regulatory pathways, respectively, and the data from qRT-PCR were consistent with that obtained from RNA-seq. In summary, PSB may be involved in the regulation of nitrogen and phosphorus nutrition, glutaminase synthesis, and abscisic acid-related metabolic pathways. This research would provide a new perspective for studying the molecular mechanism of potato growth promotion by PSB in the level of gene expression and related metabolic pathways in potato roots under the application of *Bacillus megaterium* P68.

## 1. Introduction

Potato (*Solanum tuberosum* L.), one of the most important crops globally with an annual production of more than 350 million tons in 2020, contributes significantly to global food security.^1^ Phosphorus (P) is required in high levels for potato production as it helps stimulate the growth of tubers and roots (Chea et al., 2021). As a crucial component for potato growth, P, however, is poorly available because the shallow root system of potatoes, and more than 40% of the world’s arable land is deficient in effective phosphorus (Dechassa et al., 2003; Balemi and Negisho, 2012), and available phosphorus content (AP) makes up only 0.1 to 0.5% of the total phosphorus (TP) in the soil solution (<10 μM) (Bhat et al., 2017; Islam et al., 2019). Currently, in order to keep the crop production, chemical fertilizers are mainly applied, yet most of the P recovery from chemical P fertilizers applied to crops does not exceed 25%, and 10–15% of the phosphorus fertilizer oftenly applied (Roberts and Johnston, 2015). Increased application of chemical fertilizers may cause problems such as soil caking, and metallic cations like Ca^2+^, Mg^2+^, Fe^3+^, and Al^3+^ may bind inorganic P and deposit it in the soil, making it unavailable to plants (Ma et al., 2011; Onodera et al., 2020; Xiong et al., 2020). Therefore, it is necessary to find a way to increase the AP in the soil.

Phosphate-solubilizing bacteria (PSB) release organic acids, enzymes, protons, and etc., which can dissolve insoluble phosphorus in the soil by lowering the pH of the medium for plant uptake and usage, such as gluconic acid, phosphatase, and carbonic acid, which can dissolve insoluble phosphorus. As PSB synthesizing various phytohormones necessary to encourage plant growth, minimizing the use of chemical phosphorus fertilizers, it is essential for the sustainable agricultural development (Panda et al., 2016; Estrada-Bonilla et al., 2021). Yanez-Ocampo et al. (2020) isolated a strain of *Bacillus pumilus* A3 from potato rhizosphere soil with phosphorus-solubilizing capacity of 350 mg·L^−1^ and used it in an *in vitro* culture trial of potato microplants. The data showed that A3 significantly increased the length of stem and root, and fresh weight of potato. Hanif et al. (2020) isolated a *Serratia plymuthica strain* KPS-10 from potato rhizosphere soil, with phosphorus-solubilizing capacity of 128.5 mg·L^−1^, which could significantly increase the fresh and dry weights and root biomass of potato. Chea et al. (2021) found that P deficiency reduced the mineral concentration, biomass of potato plants, and root metabolite. Under the condition of P deficiency, PGPR improved the root biomass, total root length, and root surface area. Therefore, it is worth studying the effect of PSB on the growth of potatoes reduce the use of chemical fertilizers and improve nutrient utilization in potatoes.

Compared to the DNA-seq, RNA-Seq technology can reduce errors and provide a larger dynamic range of detection. In addition, transcriptome second-generation sequencing technology is widely used for transcript mapping research for its sensitivity, resolution, unrestricted ability to detect unknown genes, and the ability to accurately identify various shear sites. At present, there are few transcriptomic studies on the interaction between PSB and potato, and the mechanism of the growth-promoting effect of PSB on potato is still unclear. Therefore, in this study, a strain of *Bacillus megaterium* P68 with P-solubilizing ability was isolated, and its growth-promoting effect on potato plants was verified by using pot tests and field trials, and transcriptomic analyses were further conducted on the root systems of potted potatoes. This would help to better explain the growth-promoting effect of *Bacillus megaterium* P68 on potato plants and would provide a theoretical basis for the application of P68.

## 2. Materials and methods

### 2.1. Screening of PSB

Isolation soil samples were collected from the rhizosphere soil of crops in South China Farm, Guangzhou, China (23° 08′N 113° 16′E). Weighed 10.0 g of isolation soil samples and placed in a 250 mL triangular flask with 90 mL sterile water (containing 5–7 glass beads), shaken for 30 min at 180 r·min^−1^ to disperse the soil samples. 5 mL of supernatant was taken in a tube and heated at 90°C for 10 min. 1 mL of the supernatant was added to a 100 mL triangular flask containing 25 mL of NBRIP medium (glucose 10.0 g, Ca_3_ (PO_4_)_2_ 5.0 g, MgCl_2_·6H_2_O 5.0 g, MgSO4·7H_2_O 0.25 g, KCl 0.20 g, (NH_4_)_2_SO_4_ 0.10 g, distilled water 1,000 mL, pH 7.0) and placed in a shaker for enrichment at 180 r·min^−1^ and temperature of 30°C. After 7 days of incubation, the supernatant of the culture was centrifuged at 6,000 r·min^−1^ for 10 min, and the orthophosphate content was determined by using the molybdenum blue method (Murphy and Riley, 1962). The medium of orthophosphates content of more than 200 mg·L^−1^ was selected and purified, the purified colonies were preserved by using 50% glycerol and kept at −80°C for further study (Kobus, 1962; Nautiyal, 1999; Xiong et al., 2015).

### 2.2. Identification of PSB

The identification of PSB was performed by evaluating the 16S rRNA gene sequence. Strains on Luria-Bertani agar medium (LB) were incubated for 18 h at 37°C, and a single colony from the plate was picked and placed in a centrifuge tube containing 100 μL of sterile water, and then heated in a water bath at 95°C for 15 min to rupture the cells and release DNA. Amplification was carried out by PCR using two universal primers, namely 27F and 1492R (Morono et al., 2014; Yoon et al., 2017), The PCR products were purified and sequenced by Tsingke Biotechnology Co., Ltd. The comparison of the sequence similarity was performed using Blastn, and some related species of the 16S rRNA sequence were downloaded and aligned using Mega7.0. The unrooted tree was constructed using the Neighbor-Joining method with a bootstrap value of 1,000 replicates (Xiong et al., 2015).

### 2.3. Potato field experiment design and yield and quality determination methods

**Figure 1:**
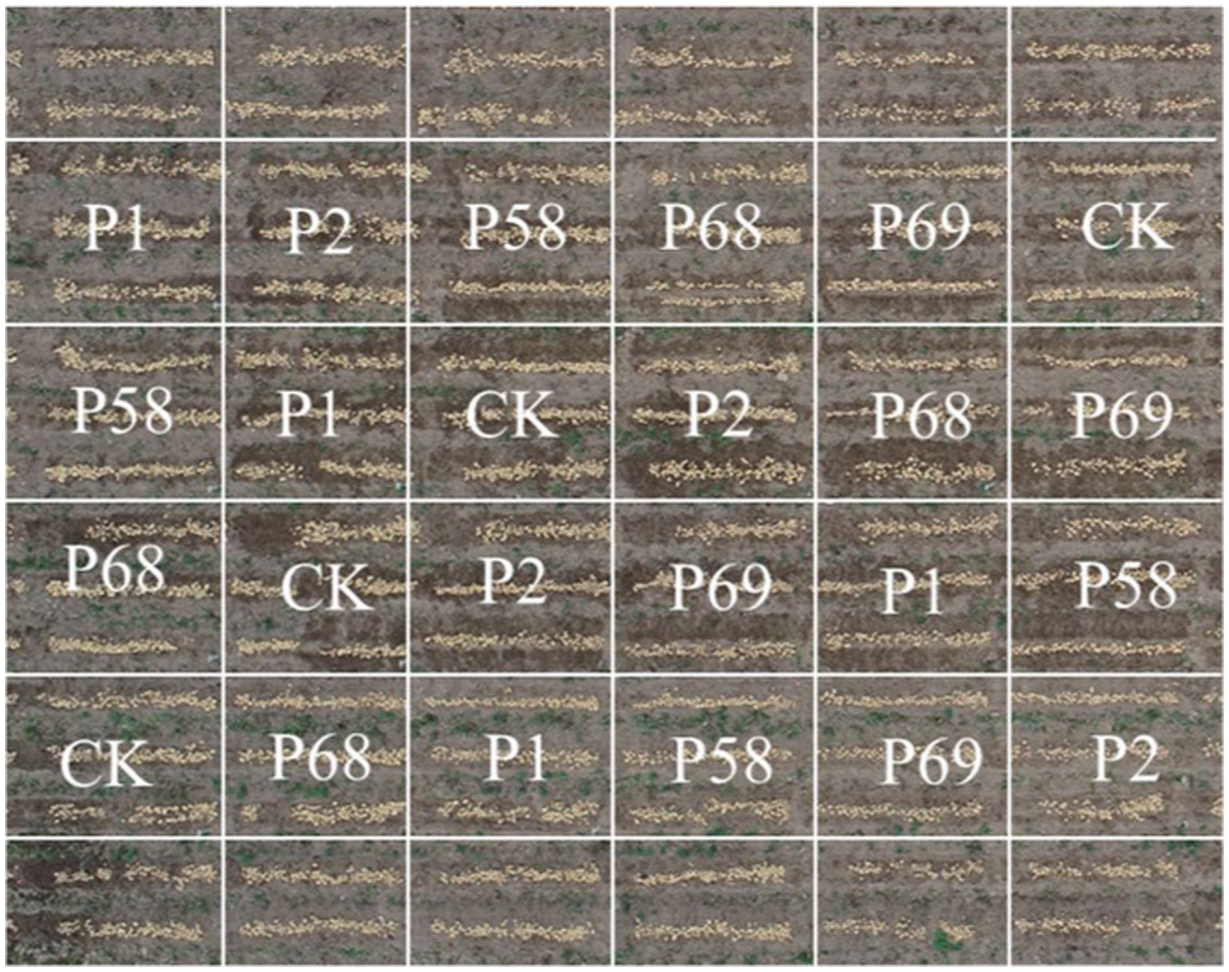
The experimental design (RCBD) graphics in field.

The trial was conducted from December 2021 to March 2022 at Zhucun Farm, Zengcheng District, Guangzhou City, Guangdong Province, China (23° 28′N 113° 71′E). Fertilizers were applied according to the recommend dose (6 t·ha^−1^) of commercial organic fertilizer, 6 t·ha^−1^ of compound fertilizer (13-6-24). Potato cultivar “*Favorita*” was used in the present study. The weight of potato blocks is about 25–30 g and each block contained 1–2 sprouts. Four replications were set up in a completely randomized block design (RCBD) with a plot area of 15.84 m^2^, divided into three monopolies with a width of 1.2 m and a length of 4.4 m (Figure 1). Two rows of 20 potato plants were planted at the spacing of 20 cm and a planting depth of 5–6 cm in each row.

The trial consisted of six treatments, control group (CK) with diatomaceous soil without bacteria agent and P1, P2, P58, P68, and P69 (isolated from the rhizosphere soil of soybean) with diatomaceous soil containing 10^8^ cfu·g^−1^ of PSB, respectively. Cfu count was determined based on the enzymatic method described by Djuuna et al. (2022): 1 g mixture was transferred using a sterile pipette to 9 ml of sterile distilled water in a test tube and stirred for 10 s to form a 10^−2^ dilution. Serial dilutions to 10^−7^ were then prepared using the same method. A 0.1 mL of aliquot from each serial dilution was transferred to a sterile LB plate and incubated at 37°C for 24 h. The number of viable cells was calculated using the following formula:

Number of cells·g^−1^ (cfu·g^−1^) = (number of colonies) × (dilution factor) (Djuuna et al., 2022) After 100 days, the yield (commercial tubers were ≥ 75 g) and quality (vitamin C, protein, reducing sugars, starch content, and the accumulation of N, P, and K) of potatoes were measured. Protein content was determined by the Coomassie brilliant blue G-250 colorimetric method with bovine serum albumin as the standard; vitamin C content was measured by the 2, 6-dichloroindophenol titrimetric method (Sha et al., 2022); reducing sugar content was evaluated by Dinitrosalicylic Acid Reagent method (Khatri and Chhetri, 2020); and starch content was determined based on the enzymatic method described by Khabou et al. (1996).

The yield was determined by weighing the tubers from the harvested rows and calculating the weight per hectare, and the percentage of dry weight was determined after drying the fresh sample at 105°C for 48 h (Samet et al., 2022). Plant material aliquot was digested in a mixture of sulfuric acid (H_2_SO_4_) and hydrogen peroxide (H_2_O_2_). The total phosphorus concentration was measured by a colorimetric assay of the P in the digest according to the Mo-Sb colorimetric method (Miquel et al., 2004; Dong et al., 2020). Total nitrogen concentration was determined by the Kjeldahl method, whereas potassium using ICP-OES (Jiang et al., 2014; Czekała et al., 2020). The N, P, and K accumulation was calculated using data on yield and N, P, and K content.

### 2.4. Potato pot experiment design

The test strain P68, which has been demonstrated with a good effect on potato yields in field trials. The soil used was taken from South China Farm (23° 08′N 113° 16′E) and the basic physical and chemical properties of the soil were: organic matter content: 13.10 g·kg^−1^, total nitrogen content (TN): 1.0 g·kg^−1^, total phosphorus content (TP): 0.98 g·kg^−1^, and available phosphorus content (AP): 27.89 mg·kg^−1^, pH 6.4.

P68 was mixed with 25 g of diatomaceous soil to make a microbial inoculum containing 10^8^ cfu·g^−1^ of cells, and CK with diatomaceous soil without bacteria was set aside. The potatoes (*Favorita*) were germinated in an Artificial Climate Chamber (temperature being 20°C for 16 h and 18°C for 8 h) until the shoots were as long as about 1 cm, cut into uniformly sized pieces with one shoot and mixed with CK and P68 immediately after the cutting. Two pieces of potatoes were planted in each pot (with height of 11.5 cm, diameter 12.5 cm, and contains 0.9 kg unsterilized soil from South China Farm) at a depth of about 6 cm, with 6 replicates of each treatment.

On the 30th day after seedlings emerged, the plant height, stem thickness, plant fresh weight, dry weight and total phosphorus content of the plant in potato were weighed and sampled. The TP and AP content of the soil was analyzed using methods described in previous assays (Watanabe and Olsen, 1965; Raun et al., 1987; Rashid et al., 2016). The potato roots were washed with sterile water three times, blotted dry with sterile filter paper, wrapped in tinfoil, labeled and frozen in liquid nitrogen immediately, and then stored at −80°C for subsequent RNA extraction. Three independent biological replicates of each sample were taken. The samples were sorted and sent to Nanjing Paisano Gene Technology for RNA extraction and transcriptome sequencing.

### 2.5. RNA extraction, library construction, and sequencing

Total RNA was extracted with Trizol Reagent (Invitrogen Life Technologies), and the concentration, quality and integrity of the RNA were determined by using a NanoDrop spectrophotometer (Thermo Scientific). The mRNA was purified from total RNA by using poly-T oligo-attached magnetic beads. Fragmentation was carried out with divalent cations under elevated temperature in an Illumina proprietary fragmentation buffer. After adenylation of the 3′ ends of the DNA fragments, Illumina PE adapter oligonucleotides were ligated to prepare for hybridization. To select cDNA fragments of the preferred 400–500 bp in length, the library fragments were purified by using the AMPure XP system (Beckman Coulter, Beverly, CA, United States). DNA fragments with ligated adaptor molecules on both ends were selectively enriched with Illumina PCR Primer Cocktail in a 15 cycle PCR reaction. Products were purified (AMPure XP system) and quantified by use of the Agilent high sensitivity DNA assay on a Bioanalyzer 2100 system (Agilent). The sequencing library was then sequenced on NovaSeq 6000 platform (Illumina) in Shanghai Personal Biotechnology Cp. Ltd.

### 2.6. Transcriptome analysis

#### 2.6.1. Quality control and differential expression analysis

Samples were sequenced on the platform to get image files, which were transformed by the sequencing platform software, and the original data in FASTQ format (Raw Data) was generated. Cutadapt (v1.15) software was used to filter the sequencing data to get high-quality sequence (Clean Data) for further analysis. The reference genome and gene annotation files were downloaded from genome website.^2^ The filtered reads were mapped to the reference genome by using HISA T2 v2.0.5.

The differences in the gene expressions were analyzed by DESeq (1.39.0) with screened conditions as follows: expression difference multiple |log2FoldChange| > 1, significant *p* < 0.05. At the same time, R language Pheatmap (1.0.8) software package was used to perform bi-directional clustering analysis of different genes of samples. The heat map was got according to the expression levels of the same gene in different samples and the expression patterns of different genes in the same sample with Euclidean method to calculate the distance and Complete Linkage method to cluster.

#### 2.6.2. GO and KEGG enrichment analysis

All the genes were mapped to Terms in the Gene Ontology (GO) database and the number of differentially enriched genes was calculated in each Term. TopGO (2.40.0) was used to perform GO enrichment analysis on the differential genes, and *p* was calculated by the hypergeometric distribution method (the standard of significant enrichment is *p* < 0.05), and the GO term with significantly enriched differential genes was found to determine the main biological functions performed by differential genes. ClusterProfiler (3.16.1) software was used to carry out the enrichment analysis of the Kyoto Encyclopedia of Genes and Genomes (KEGG) pathway of differential genes, focusing on the significant enrichment pathway with *p* < 0.05.

### 2.7. Quantitative real-time polymerase chain reaction analysis

The expression levels of genes in the RNA sequencing results were determined by performing qRT-PCR analyses on six selected DEGs. The primers used were designed with Primer 5.0 (Supplementary Table S1). A total of 800 ng of RNA was used to synthesize cDNA. 1 μL gDNA Remover and 1 μL 10 × gDNA Remover Buffer, and then were added into the RNA-free tube with ice bath. The volume of RNase-free double-distilled H_2_O was fixed to 10 μL. The reaction mixture was gently mixed, centrifuged for 3–5 s, centrifuged at 60°C for 5 min, immersed in ice bath for 2 min, and centrifuged again for 3–5 s. The following reagents were added to the test tube with ice bath: 4 μL of 5× RT Reaction Mix, 1.0 μL of SynScriptTM III RT Enzyme Mix (SynScriptTM III cDNA Synthesis Mix, China), and fixed to 20 μL use RNase-free double-distilled H_2_O. The mixture was gently mixed and centrifuged for 3–5 s. The reverse transcription reaction was carried out on the PCR instrument at 25°C for 10 min, 50°C for 15 min, and 85°C for 5 min. The mixed solution was stored at −20°C. The cDNA sample was diluted 10 times as the template for on-board detection. Three technical replicates for each sample were obtained for qRT-PCR, which was run at 95°C for 1 min and then at 40 cycles each of 95°C for 10 s and 60°C for 30 s. The EF-1α gene was used as the control. A real-time PCR system (QuantStudio 6 Flex, Biosystems) was used for qRT-PCR.

### 2.8. Statistical analysis

Statistical analysis was performed with Microsoft Excel 2013 and IBM SPSS Statistics 26. Duncan’s test and independent-samples *t*-test (*p* < 0.05) were used for significance testing.

## 3. Results

### 3.1. The screening and identification of PSB

Strains of with P-solubilizing ability were isolated from the soybean rhizosphere soil at the South China Agricultural University farm (23° 08′N 113° 16′E), and those strains with better growth-promoting ability being conserved. After 7 days of incubation in NBRIP medium, the strains P1, P2, P58, P68, and P69 had higher P-solubilizing ability, and P-solubilizing capacity of 392.56, 296.77, 371.16, 461.86, and 634.57 mg·L^−1^, respectively. Morphological observation of these strains was carried out on LB medium after 24 h of growth. The strain was observed under microscopy as gram-positive, spore-producing bacteria with rod-shaped cells (Supplementary Figure S1).

Through 16S rRNA sequencing, PSB P1, P2, P58, P68, and P69 were identified as *Bacillus*. Among them, P68 was identified as *Bacillus megaterium* with 99% homology (Supplementary Figure S2), upload P68 16S rRNA sequencing to the GenBank database and get the GenBank accession number is OQ391206.

### 3.2. PSB improved potato yield and quality In field

Compared with the CK, the treatment with microbial inoculum made of PSB promoted the potato yield of commercial tubers (Table 1). PSB increased the yield of small tubers and commercial tubers by 3.00–18.92% and 9.59–17.02%, respectively. According to the market purchase price of potatoes (236.17 dollar·t^−1^), the use of PSB can increase the income of 825.24–1462.72 dollar·ha^−1^. *Bacillus megaterium* P68 with the best effect increased the yield of small tubers and commercial tubers by 8.71 and 17.02%, respectively. In addition, the use of PSB could improve the content of vitamin C by 8.48–22.89%, protein by 49.68–123.75%, and starch by −2.69–2.08%, respectively (Table 2). Meanwhile, PSB improved the accumulation of N, P, and K by −8.02−7.70%, 14.57−27.31% and 10.755−12.65%, respectively. Among those, P68 increased the content of vitamin C by 19.44%, protein by 67.78%, and starch by 1.29%, increased N accumulation by 3.87%, P accumulation by 27.31% and K accumulation by 12.54% (Table 3).

**Table 1:** Effect of PSB on potato field yield.

	Small tuber (t·ha^−1^)	Commercial tuber (t·ha^−1^)	Increment (dollar·ha^−1^)
CK	3.33 ± 0.34 a	36.39 ± 1.76 b	/
P1	3.96 ± 0.37 a	40.86 ± 1.34 a	1055.69
P2	3.75 ± 0.34 a	39.88 ± 1.23 ab	825.24
P58	3.50 ± 0.33 a	40.17 ± 1.42 ab	893.82
P68	3.62 ± 0.30 a	42.58 ± 0.86 a	1462.72
P69	3.43 ± 0.25 a	40.20 ± 0.71 ab	901.17

Commercial tuber refers to the weight of potato tuber ≥ 75 g, Small tuber refers to the weight of potato tuber < 75 g, the increment is calculated according to the market price of 236.17 dollar·*t*^−1^. Data are expressed as means ± standard error (*n* = 4), and different letters indicate significant differences between different treatments at *p* < 0.05 in Duncan’s test.

**Table 2:** Effect of PSB on potato quality.

	Vitamin C (mg·kg^−1^)	Protein (g·100 g^−1^)	Reducing sugar (%)	Starch (%)
CK	190.73 ± 8.78 a	0.78 ± 0.16 c	0.10 ± 0.01 a	17.83 ± 1.13 a
P1	234.38 ± 10.69 a	1.29 ± 0.05 b	0.15 ± 0.01 a	18.72 ± 0.5 a
P2	206.90 ± 4.89 a	1.48 ± 0.16 ab	0.12 ± 0.02 a	17.85 ± 0.64 a
P58	207.50 ± 14.32 a	1.74 ± 0.11 a	0.15 ± 0.02 a	17.35 ± 0.71 a
P68	227.8 ± 30.65 a	1.31 ± 0.01 b	0.09 ± 0.00 a	18.06 ± 0.44 a
P69	220.83 ± 13.82 a	1.17 ± 0.19 bc	0.12 ± 0.02 a	17.96 ± 1.36 a

Data are expressed as means ± standard error (*n* = 4), and different letters indicate significant differences between different treatments at *p* < 0.05 in Duncan’s test.

**Table 3:** Effect of PSB on the accumulation of N, P, and K in potato tubers.

	N accumulation (kg/666.67m^−2^)	P accumulation (kg/666.67m^−2^)	K accumulation (kg/666.67m^−2^)
CK	77.10 ± 1.13 a	11.09 ± 0.62 a	157.26 ± 4.73 a
P1	70.92 ± 7.22 a	13.14 ± 0.34 b	174.59 ± 9.71 a
P2	79.93 ± 2.92 a	13.52 ± 0.77 b	174.17 ± 5.34 a
P58	83.03 ± 3.76 a	13.58 ± 0.42 b	177.15 ± 8.97 a
P68	80.08 ± 5.57 a	14.11 ± 0.62 b	176.98 ± 9.81 a
P69	76.65 ± 4.23 a	12.70 ± 0.62 ab	175.78 ± 3.74 a

Data are expressed as means ± standard error (*n* = 4), and different letters indicate significant differences between different treatments at *p* < 0.05 in Duncan’s test.

### 3.3. P68 Promotes the growth of potato plants in pot experiment

**Figure 2:**
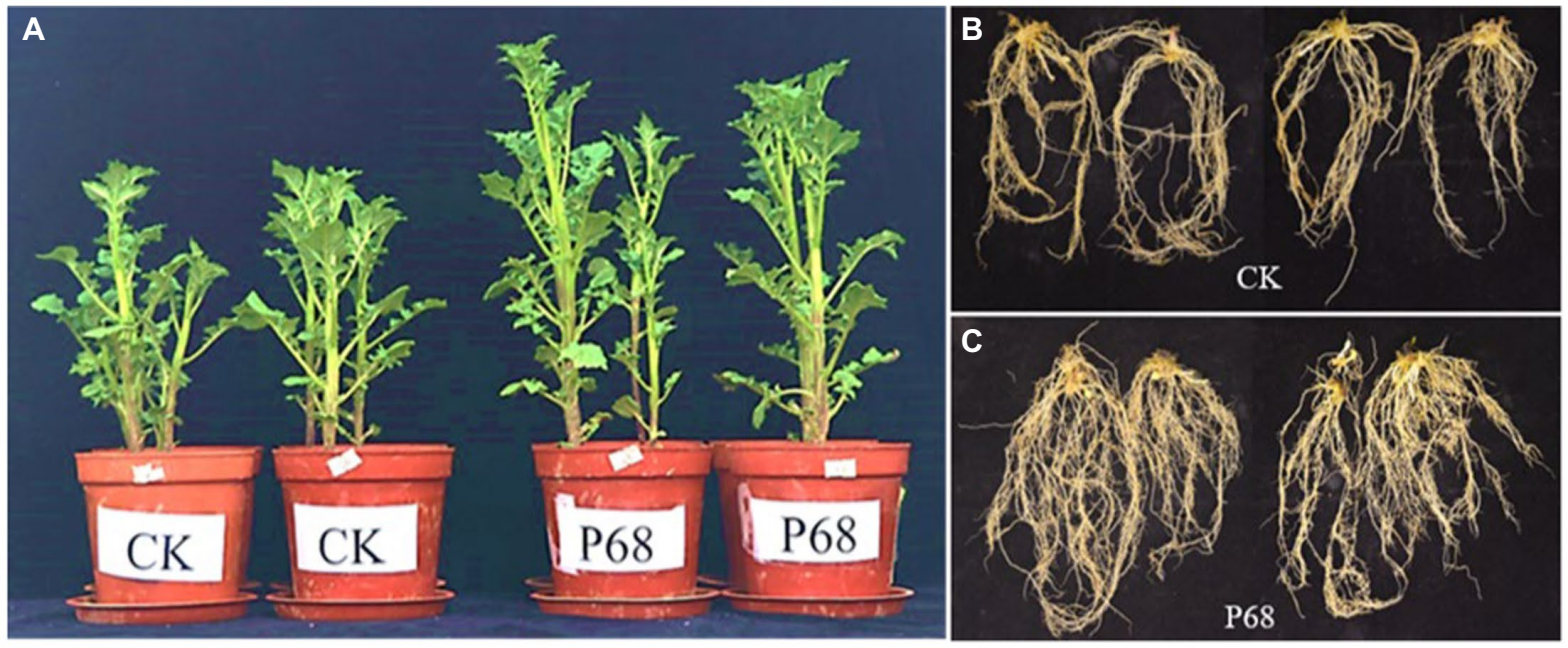
Promoting effect of P68 on potato plants in pot experiment. **(A)** Compared with CK, P68 significantly promoted plant growth on the 30 days after planting. **(B)** Roots of plants in CK treatment group. **(C)** Roots of plants in P68 treatment group.

Based on the results of the field experiment, the effect of PSB P68 on potato growth was investigated after 30 days of planting in pot experiment (Figure 2). As listed in Table 4, the application of P68 significantly increased the height, stem thickness, fresh and dry weights of stem and leaf, length, fresh and dry weights of roots, and TP content of the plants by 35.05, 22.25, 32.33, 31.28, 8.16%, 69.54%, 63.64, and 37.50%, respectively. And the root length of CK and P68 are almost consistently, but significantly increased the dry weight of roots, and compared with CK, the root structure of P68 was intensively tighter, and have more fibrous roots under P68 treated potato (Figure 2). In addition, the application of P68 decreased the pH of the soil and increased available phosphorus (AP) content of the soil by 29.15%.

**Table 4:** Growth condition of potato in P68 treatments.

Trement	CK	P68
Height/cm	21.83 **±** 0.96	29.48 ± 0.56*
Stem thickness/mm	6.83 ± 0.42	8.35 ± 0.31*
Fresh weight of stem and leaf/g	16.64 ± 0.72	22.02 ± 0.53*
Dry weight of stem and leaf/g	1.79 ± 0.11	2.35 ± 0.07*
Root length/cm	22.68 ± 3.35	24.53 ± 2.30 ns
Fresh weight of root/g	3.22 ± 0.65	5.46 ± 0.69*
Dry weight of root/g	0.44 ± 0.05	0.72 ± 0.05*
TP of plant/%	0.32 ± 0.02	0.44 ± 0.01*
AP of soil/(mg/kg)	26.96 ± 0.73	34.82 ± 1.63*
pH of soil	5.98 ± 0.02	5.89 ± 0.06 ns

Data are expressed as means ± standard error (*n* = 4), and *indicate significant differences between different treatments at *p* < 0.05 in Independent-samples *t*-test.

### 3.4. Analysis of gene transcript levels in potato roots inoculation with P68

#### 3.4.1. Transcriptomic analysis using Illumina-based RNA sequencing

Three samples each from the two treatments (CK and P68) were subjected to RNA sequencing (Table 5). The sequenced results showed that the total number of bases was about 6G (not less than 95% of 6G), and Q30 (%) was 92.35–94.8%. In addition, some low-quality Reads with joints were filtered out from the data, and the percentage of high-quality sequence bases in sequencing bases was between 93.84–94.05%, all of which were high and stable. It indicates that the sequencing results are stable and reliable. In addition, the total and single alignment rates of the sequencing results were higher than 80 and 90%, respectively, with high and stable alignment rates, while the multiple alignment rates were lower and stable, both lower than 6%, indicating that the sample had a high matching degree with the selected reference genome, which could be used for subsequent reference transcriptome analysis.

**Table 5:** Summary of sequencing results and comparison results.

Sample	CK-1	CK-2	CK-3	P68-1	P68-2	P68-3
Bases (G)	6.59	6.09	5.99	6.11	5.66	6.78
Q30(%)	93.98	94.35	94.8	94.48	92.35	94.3
Clean Data (G)	6.19	5.72	5.64	5.74	5.32	6.36
Clean Data (%)	93.94	93.95	94.05	94.02	93.96	93.84
Total Mapped	31,999,004 (83.58%)	29,772,238 (84.50%)	37,416,062 (88.22%)	35,910,354 (87.64%)	32,711,106 (85.72%)	31,836,414 (84.70%)
Multiple Mapped	1,557,291 (4.87%)	1,680,201 (5.64%)	1,548,476 (4.14%)	1,494,275 (4.16%)	1,338,507 (4.09%)	1,756,992 (5.52%)
Uniquely Mapped	30,441,713 (95.13%)	28,092,037 (94.36%)	35,867,586 (95.86%)	34,416,079 (95.84%)	31,372,599 (95.91%)	30,079,422 (94.48%)

Bases(G): the total number of bases; Q30(%): the percentage of bases with base recognition accuracy above 99.9%; Clean Data (G): base number of high-quality sequences; Clean Data %: percentage of high-quality sequence bases in sequencing bases; Total Mapped: Total sequence Mapped/Clean Reads for the reference genome; Multiple Mapped: Total number of sequences Mapped to Multiple locations. Percentage is multiple Mapped/total mapped; Uniquely Mapped: Total number of sequences aligned to one position only, percentages are Uniquely Mapped/total Mapped.

#### 3.4.2. Biological links between differential gene expressions

**Figure 3:**
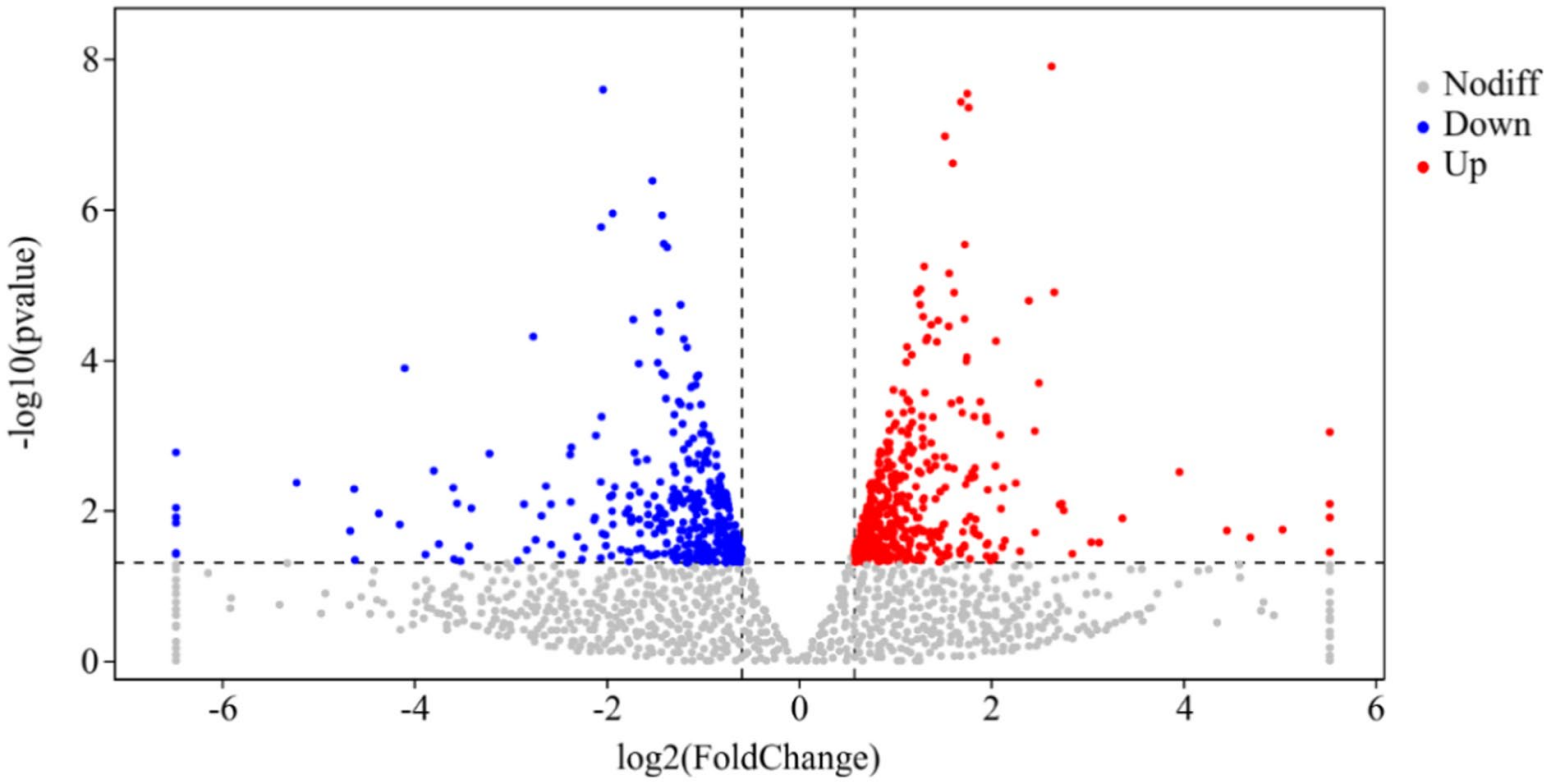
Volcanic map of differentially expressed genes of CK and P68. The *X*-axis is the log2 of the Fold Change, and the *Y*-axis is the value of the −log_10_ at the significance level. The two dotted lines in the figure represent the threshold values of the difference multiples. The dotted line is the threshold of significance level. Red dots indicating upregulated genes in this group; blue dots indicating downregulated genes, and gray dots indicating non-significantly differentially expressed genes.

**Figure 4:**
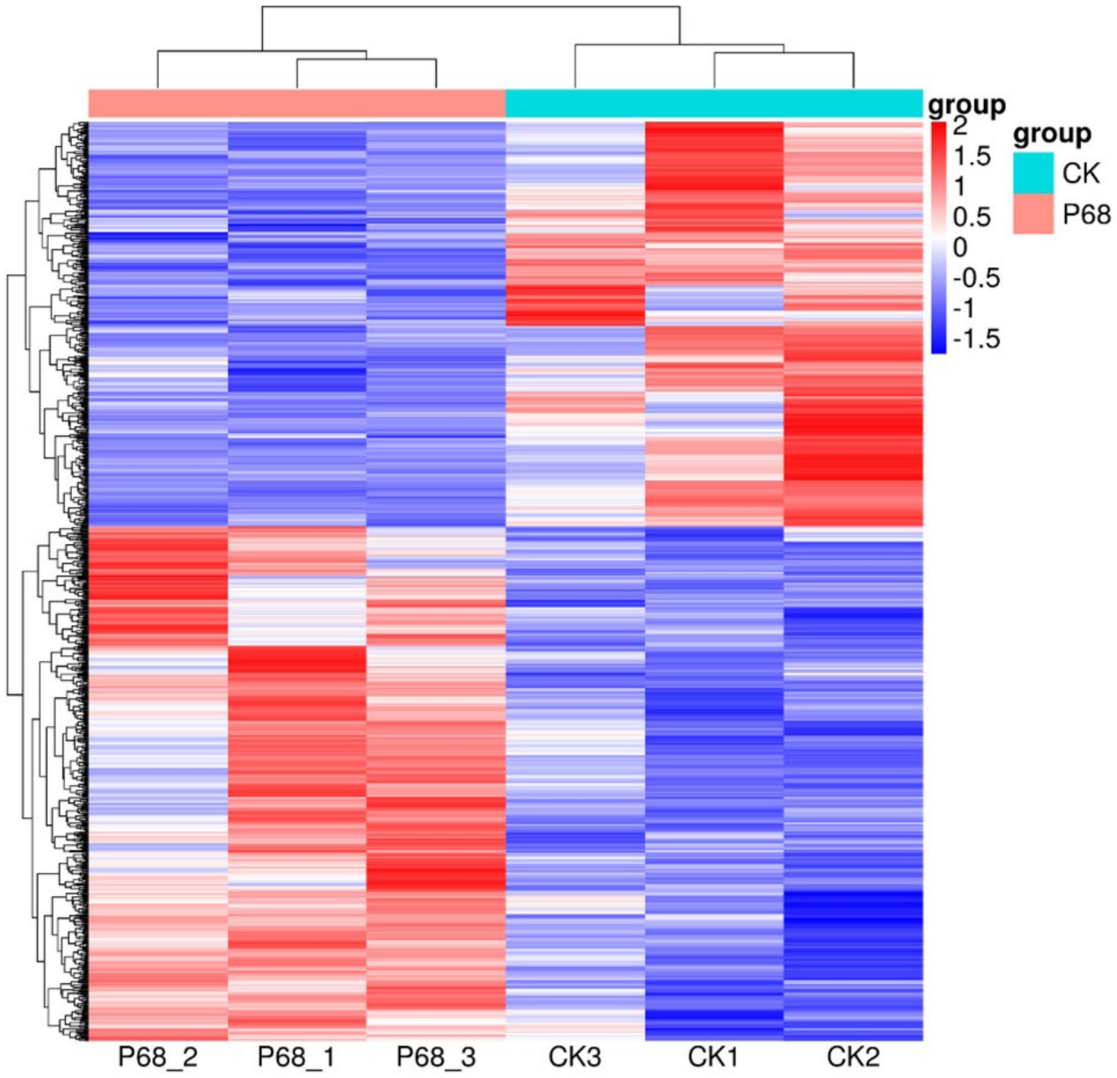
Cluster analysis diagram of differentially expressed genes of CK and P68. Each row represents a gene, and each column represents a sample (P68-1, P68-2, and P68-3 represent three replicates of P68 sample. CK1, CK2, and CK 3 represent three replicates of CK sample). Different colors in the clustering heat map represent the different expression levels of genes. Red indicates high-expression genes and blue indicates low-expression genes, respectively.

DESeq was used for differential analysis of gene expressions, compared with the CK, 784 DEGs were expressed in the P68 treated potato roots, including 439 upregulated genes and 345 downregulated genes. The R ggplots2 software was used to draw a volcanic map of differentially expressed genes to depict the gene distribution, gene expression with multiple differences, and significance results (Figure 3). Cluster analysis was used to determine the expression patterns of differentially expressed genes under different experimental conditions, genes with high-expression correlations between samples were often grouped together, these genes were linked in some biological process, or a metabolic or signaling pathway (Figure 4). Thus, through expression clustering analysis (Figure 4), it was found that genes with unknown biological connections were linked to each other.

#### 3.4.3. P68 affects potato growth through energy, amino acid, and carbohydrate metabolic pathways

**Figure 5:**
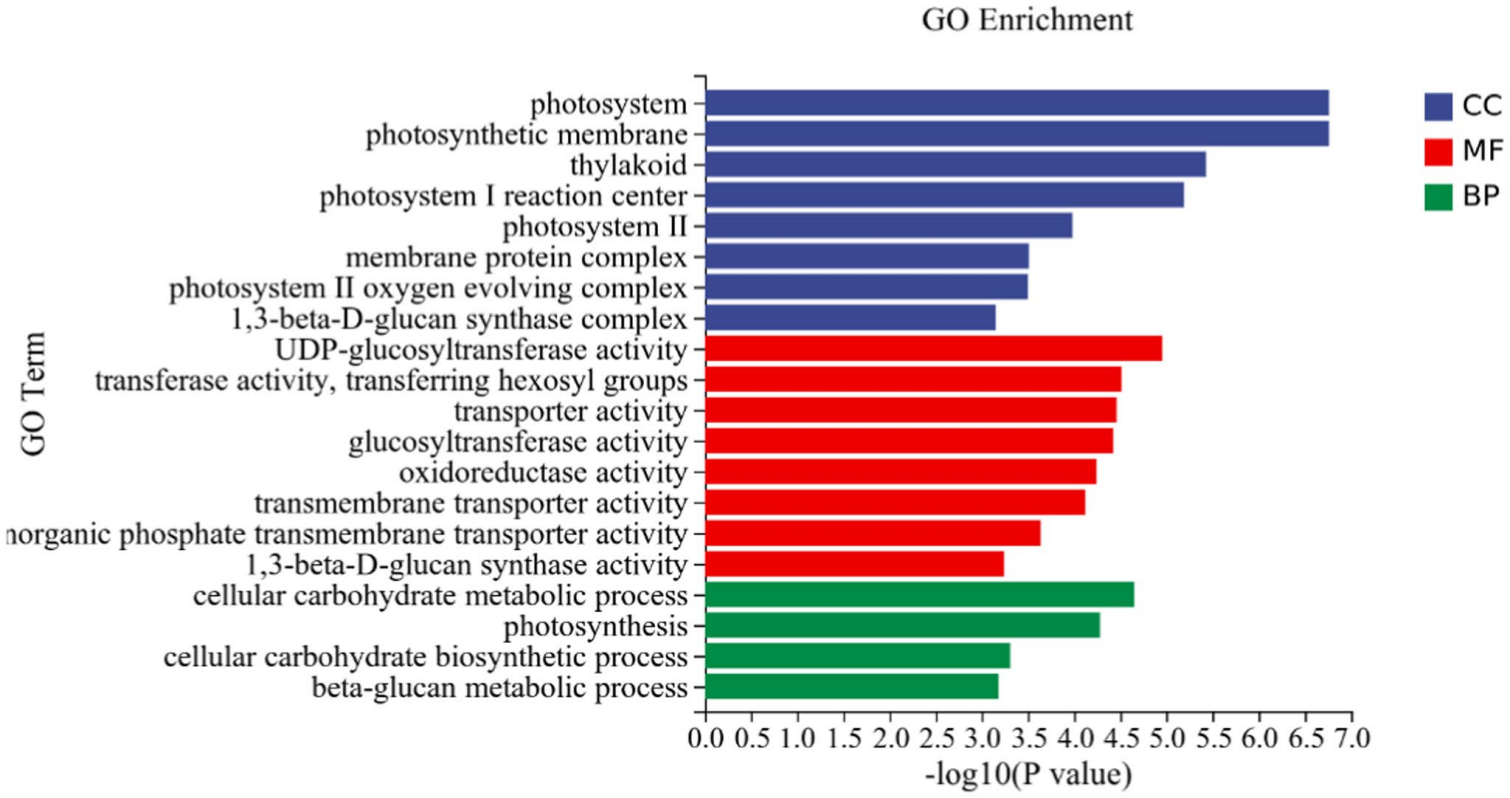
DEGs in potato tuberous of CK and P68. Top 20 pathways with the most significant enrichment in GO term between CK and P68. The *X*-axis is GO term, indicates the number and percentage of DEGs under each functional classification, whereas the *Y*-axis is GO term enriched -log10 (*p*-value), represents the enriched GO functional classification, which is divided into three categories: cellular component, molecular function and biological process.

GO analysis: In this experiment, 8,450 DEGs were enriched in the GO classification of potato roots treated with P68 compared with CK, and 1,082 functional entries were enriched, of which 2,510 DEGs were enriched in 306 functional entries in Molecular function (MF). In Cellular component (CC), 1,402 DEGs were enriched in 91 functional entries, and in Biological process (BP), 4,538 DEGs were enriched in 685 functional entries, of which GO enrichment analysis was performed by using topGO (*p* < 0.05). The top 20 GO entries with the most significant enrichment were selected in the three broad categories of CC, MF, and BP and presented by the histogram (Figure 5). The genes expressed quite differentially in potato roots by use of P68 were enriched in CC particularly in membrane protein complex (GO:0098796). In MF, they were mainly enriched in UDP-glucosyltransferase activity (GO:0035251), transferase activity, transferring hexosyl groups (GO:0016758), transport activity (GO:0005215), glucosyltransferase activity, oxidoreductase activity (GO:0016491), transmembrane transporter activity (GO:0022857), inorganic phosphate transmembrane transporter activity (GO:0005315), and 1, 3-beta-D-glucan synthase activity (GO:0003843). The DEGs in BP were mainly enriched in the cellular carbohydrate metabolic process (GO:0044262), photosynthesis (GO:0015979), cellular carbohydrate biosynthetic process (GO:0034637), and beta-glucan biosynthetic process (GO:0051273). And in the GO factor diagram, DEGs were mainly distributed in MF with respect to transferase activity, transferring hexosyl groups (GO:0016758), transporter activity (GO:0005215), oxidoreductase activity (GO:0016491), and transmembrane transporter activity (GO:0022857; Figure 6).

**Figure 6:**
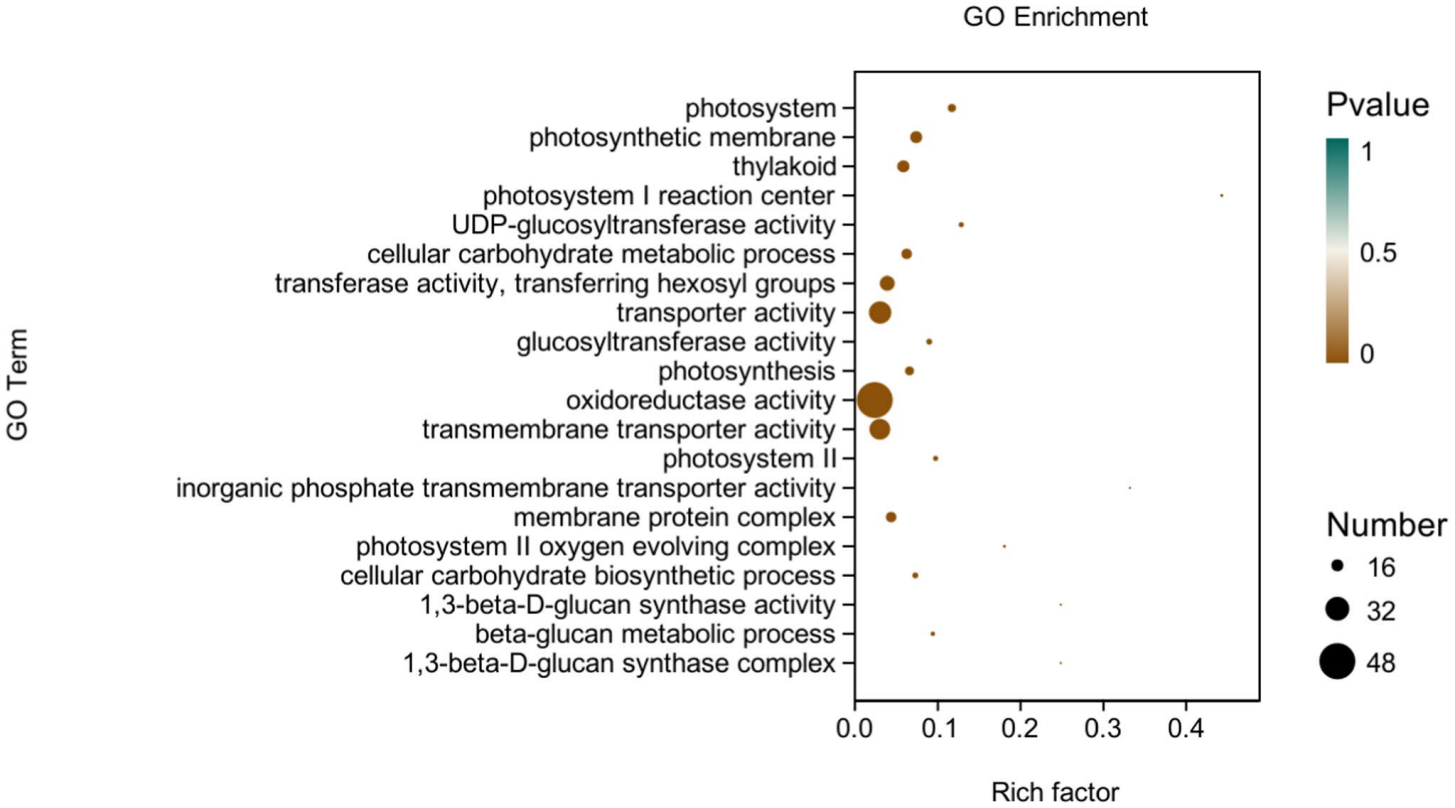
GO-enriched pathways of CK and P68. The *X*-axis is rich factor (the number of differential genes annotated to GO Term/the total number of genes annotated to GO Term), and the *Y*-axis is GO Term. The size of points in the figure represents the number of differential genes annotated to corresponding term (upregulated or downregulated, related to the gene set selected during analysis). The depth of the color indicates the level of significance.

**Figure 7:**
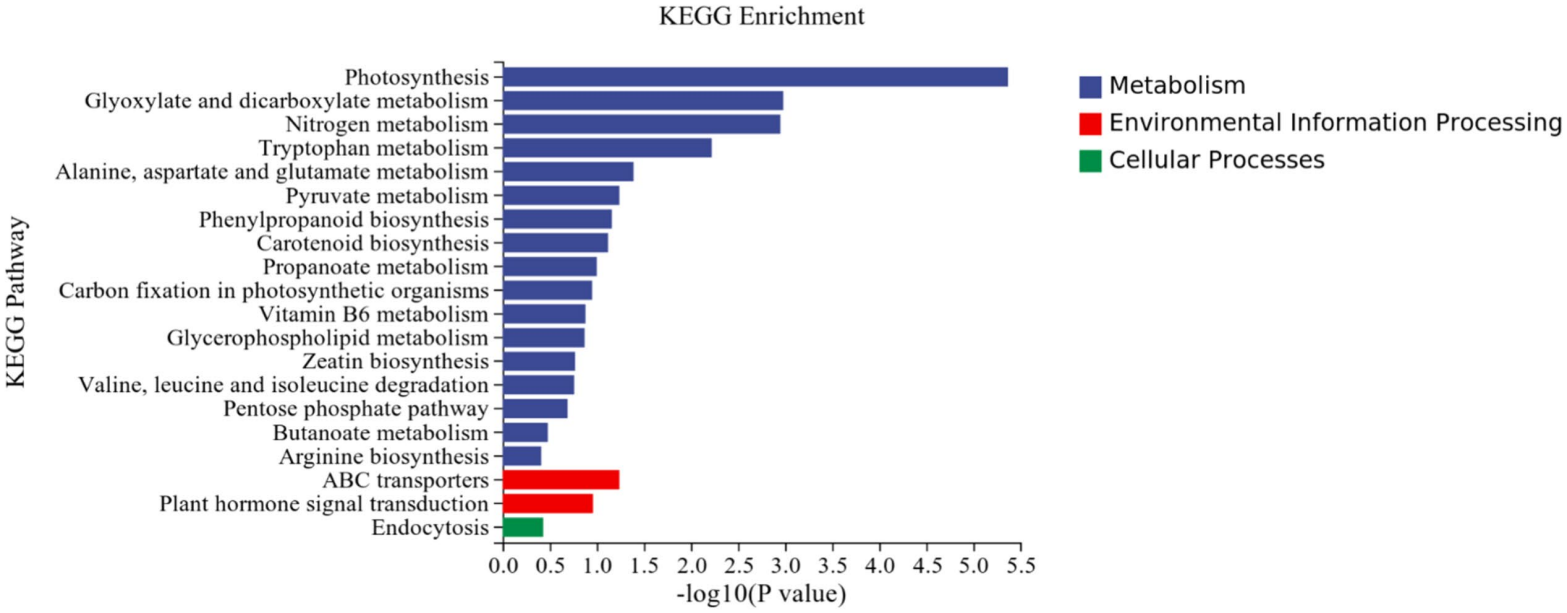
Results of KEGG enrichment analysis of CK and P68. Top 20 pathways with the most significant enrichment in KEGG pathway between CK and P68. The *X*-axis is pathway, indicates the number and percentage of DEGs under each functional classification, whereas the *Y*-axis is pathway enriched -log10 (*value of p*), represents the enriched KEGG pathway classification.

**Figure 8:**
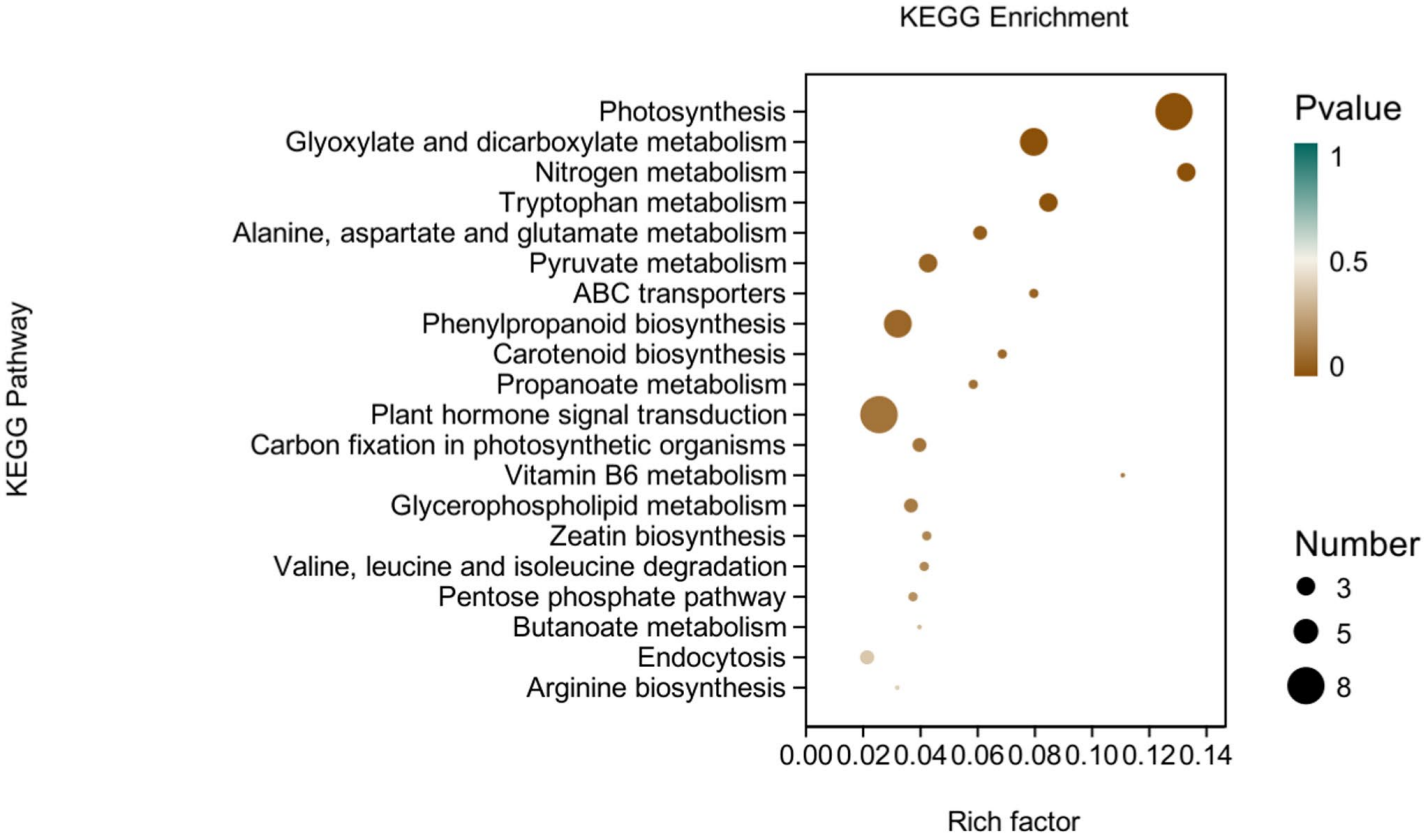
pathways enriched in KEGG analysis of CK and P68. The *X*-axis is rich factor (the number of differentially annotated genes in Pathway/the total number of genes annotated in this Pathway), and the *Y*-axis is Pathway. The size of dots in the figure represents the number of differentially annotated genes in corresponding pathway (upregulated or downregulated, related to the gene set selected for analysis). The depth of the color indicates the level of significance.

KEGG analysis: KEGG analysis plots showed that 101 DEGs were annotated to 46 categorical metabolic pathways in the KEGG database for the DEGs in potato roots were treated with P68. The 20 most significant pathways mainly involved in plant metabolic pathways were glyoxylate and dicarboxylate metabolism (sot00630), nitrogen metabolism (sot00910), tryptophan metabolism, alanine (sot00380), alanine, aspartate and glutamate metabolism (sot00250), pyruvate metabolism (sot00620), phenylpropanoid biosynthesis (sot00940), carotenoid biosynthesis (sot00906), propionate metabolism (sot00640), carbon fixation in photosynthetic organisms (sot00710), vitamin B6 metabolism (sot00750), glycerophospholipid metabolism (sot00564), zeatin biosynthesis (sot00908), valine, leucine and isoleucine degradation (sot00280), pentose phosphate pathway (sot00030), butyric acid metabolism (sot00650), arginine biosynthesis (sot00220), ABC transporter (sot02010), and plant hormone signal transduction (sot04075) of the environmental information processing pathway, and endocytosis (sot04144) of cellular processes (Figure 7). In addition, a large number of enriched genes were distributed in glyoxylate and glyoxylate and dicarboxylate metabolism (sot00630), nitrogen metabolism (sot00910), tryptophan metabolism (sot00380), alanine, aspartate and glutamate metabolism (sot00250), pyruvate metabolism (sot00620), phenylpropanoid biosynthesis (sot00940), and plant hormone signal transduction (sot04075) (Figure 8).

### 3.5. qRT-PCR analysis

In order to clarify the possible pathway by which P68 inoculation affects potato yield and quality, related genes in the pathway were analyzed according to the above KEGG and GO enrichment results. And a total of 6 genes that may interact with P68 were found for verification analysis (Supplementary Figure S3). The functional annotations of 6 genes (Supplementary Table S1) are as follows: Inorganic phosphate transporter (PGSC0003DMG400010288), Inorganic phosphate transporter (PGSC0003DMG400013451), Nitrate transporter (PGSC0003DMG400006913), Glutamine synthetase (PGSC0003DMG400004355), SAUR family protein (PGSC0003DMG400031744), and Abscisic acid receptor PYL4 (PGSC0003DMG400015897). Quantitative primers were designed according to the above 6 gene sequences, and qRT-PCR analysis was conducted. The results were consistent with the transcriptome data. The expression of gene 2,3,4,6 was significantly upregulated, while the expression of gene 1 and 5 was not significantly different, indicating that the inoculation might be related to plant hormone signal transduction and transporter activity.

## 4. Discussion

### 4.1. The screening and identification of PSB

In this study, a highly effective P-solubilizing strain was screened and identified as *Bacillus megaterium*, with a P-solubilizing capacity of 461.86 mg·L^−1^ after 7 days of incubation in NBRIP medium, which was higher than the *Bacillus* strain P1 (392.56 mg·L^−1^), P2 (296.77 mg·L^−1^), P58 (371.16 mg·L^−1^), *Bacillus subtilis* KSP-11 (66.4 mg·L^−1^) (Hanif et al., 2015), and *Bacillus pumilus* A3 (350 mg·L^−1^) (Yanez-Ocampo et al., 2020). Through 16S rRNA sequencing, P68 was identified as *Bacillus megaterium* with 99% homology (Supplementary Figure S2), and improved potato yield and quality in potato roots inoculation with P68 (Table 1). It was found that *Bacillus megaterium* can produce a large amount of organic acids in the growth and reproduction process, which can decompose or dissolve insoluble phosphorous substances in soil and convert them into phosphorus elements easily absorbed by plants, thus improving the utilization rate of phosphorus elements (Do Carmo et al., 2019). In addition, *Bacillus megaterium* rapidly propagated in the soil and became the dominant bacteria, which controlled the nutrition and other resources of the rhizosphere (Bhatt and Maheshwari, 2020). Therefore, in this study, *Bacillus megaterium* with strong phosphorus solubilization ability was selected as the inoculum in field and pot experiment.

### 4.2. The effects of PSB on potato plant growth and yields

Currently, there are two main mechanisms by which PSB solubilize phosphorus. One is the secretion of organic acids by PSB, such as malic acid and gluconic acid, which solubilize insoluble phosphorus by reducing soil pH (Scervino et al., 2010; Zhang et al., 2020). The other is to increase the effective phosphorus in the soil by secreting enzymes and other substances that can dissolve insoluble phosphorus, such as phytase, acid, and alkaline phosphatase, or by producing plant hormones, such as IAA and GA, to promote plant growth and improve plant ability to absorb and use phosphorus from the soil. PSB could increase the effective phosphorus in the soil and be of help in plant uptake, thus promoting plant growth. Mosela et al. (2022) applied PSB, increased yields by 17.8% in maize and 26.5% in soybean. Zaheer et al. (2019) isolated PSAZ17 from sandy soil and applied it to chickpea field experiment, compared with CK, it could increase grain yield by 1,127 kg·ha^−1^ and increase phosphorus content in grain by 25.69%. In our experiment, P68 have a remarkable effect on plant height, biomass, etc., increased the TP of plant and AP of the soil, and the pH of the potted soil was reduced, so it was presumed that P68 was promoting plant growth by secreting organic acids to increase the effective phosphorus content of the soil. Whereas microbial agents were affected by climate, humidity, and other factors in field trials, the strains that had an effect in the laboratory might not necessarily have a growth-promoting effect in field trials (Gyaneshwar et al., 2002; Pastore et al., 2020; Ding et al., 2021). In this study, P68 treatment promoted potato root growth, increased fibrous, reduced soil pH, and increased soil available phosphorus content (Tables 1, 4), the results of the pot and field experiments were consistent, which indicated that strain P68 promoted the growth of potato roots, possibly reduced soil pH and increased the available phosphorus content to increase potato commercial tuber in potato rhizosphere by secreting organic acids from the roots. More importantly, the application of PSB could increase the yield of commercial tubers and then increase farmer’s income. In addition, PSB could also improve the quality of the element content, such as vitamin C, protein and starch content. These results showed that both in field and laboratory environments, P68 was able to promote potato growth.

However, the P-solubilizing capacity of P68 strain was not the highest in this study, lower than *Bacillus* strain P69 (634.57 mg·L^−1^), but it showed the best growth-promoting ability. There might be other possible functions of this bacterium, such as IAA production and potassium resolution capacity, which was waiting for further exploration. Moreover, some PSB also presented a good growth-promoting effect on a variety of crops. For example, *Bacillus megaterium* could promote the growth of barley crops (Ibanez et al., 2021), tomato (Katsenios et al., 2021), and Millet (Silva et al., 2021). Thus the inoculation with PSB and other strains could enhance almost all plant growth parameters (Nacoon et al., 2020). Therefore, it would be a valuable and interesting experiment to study the effect of P68 on other plants in future, considering that co-inoculation of PSB and other strains might have better adaptability to complex rhizosphere environment. Thus, the trails of combined PSB with other stains were in great need for further study.

### 4.3. Differential enrichment analysis of gene expression in potato roots

#### 4.3.1. GO analysis

To further explain the growth-promoting effect of P-solubilizing *Bacillus* P68 on potato, this experiment was conducted through the transcriptome analysis of the root system of potato pot trials. DEGs in the presence of P68 in the potato root system, according to the GO analysis, were mainly involved in the MF aspects of transferase activity, transferring hexosyl groups (GO:0016758), transporter activity (GO:0005215), oxidoreductase activity (GO:0016491), and transmembrane transporter activity (GO:0022857). Zhang et al. (2021) reported that P-solubilizing bacteria M01 had mainly affected the synthesis, transport, and carbohydrate pathways of secondary metabolites in melon seedlings so as to enhance phosphorus uptake and utilization in melon. In addition, differential gene expression enrichment analysis of the stem and leaves of pepper, which was promoted by the action of P-solubilizing bacteria on pepper, showed that DEGs mainly focused on biological processes (BP) and other functional genes, such as oxidoreductase activity (GO: 0016491) (Zhang et al., 2020). Li et al. (2020) found that DEGs in potato were mostly enriched in functions such as transport activity and oxidoreductase activity under salt stress based on GO analysis and KEGG analysis. Similarly, the results reported by Negi et al. (2020) found that when leaves of salt-tolerant and salt-sensitive sugarcane were subjected to transcriptome analysis and showed that sugarcane under salt stress induced genes related to metabolic processes, transmembrane transport, and photosynthesis expression, to reach a steady state in sugarcane production, thus increasing sugarcane yield under salt field conditions. Overall, genes of plants under inoculating with PSB may be regulated to cope with adverse growth conditions.

#### 4.3.2. KEGG analysis

It has been reported that P-solubilizing bacteria are related to the metabolic pathways of amino acids and carbohydrates in pepper plants (Zhang et al., 2020). This is similar to this study, in which most of DEGs were involved in energy metabolism, carbohydrate metabolism, and amino acid metabolism in the effect of PSB on potato roots through KEGG metabolic pathway analysis. This was mainly through, glyoxylate and dicarboxylate metabolism (sot00630),; nitrogen metabolism (sot00910),; tryptophan metabolism (sot00380), alanine, aspartate, and glutamate metabolism (sot00250), pyruvate metabolism (sot00620), phenylpropanoid biosynthesis (sot00940), and plant hormone signal transduction (sot04075). Similarly, it has also been reported that DEGs in garlic are mainly distributed in the phenylpropanoid biosynthesis (sot00940) and plant hormone signal transduction (sot04075), which is related to both long and short daylight treatment (Atif et al., 2021). The results in this study coincided with Liu et al. (2022) who demonstrated that under the heat stress of *maize* for KEGG analysis, DEGs were mainly enriched in plant hormone signal transduction, alanine, aspartate, and glutamate metabolism, ABC transporter, and zeatin biosynthesis.

In conclusion, the interaction between P68 and potato might be linked to genes that regulated environmental drought, salt and heat stress in response to complex environments, and changes in the AP to promote plant growth. P68 strain has played an important role in promoting the energy metabolic pathway, the metabolic pathway of amino acids, and the carbohydrate metabolic pathway in potato growth. And the results of qRT-PCR showed that inoculation of P68 might be related to Inorganic phosphate transporter, Glutamine synthetase, and Abscisic acid receptor in potato plants (Supplementary Figure S3), it was possible that P68 increased the AP content of the soil. As a participant in a series of physiological metabolic activities, such as photosynthesis, respiration, and material transport, the content of AP in the soil was increased to promote root growth, improve root activeness, enhance plant ability to absorb soil nutrients, and improve plant resistance and adaptability to drought, cold and salt stress. In addition, for potato, the higher the P content in the plant in the early stages of growth, the stronger the photosynthesis and material metabolism were, and the more carbohydrates were transported from the stalk to the underground part, which facilitated tuber formation and starch accumulation (Raghothama and Karthikeyan, 2005; Poirier et al., 2022). Therefore, P68 plays an important role in increasing tuber yield and enhancing tuber quality of potato.

## 5. Conclusion

An high-efficiency phosphorus-solubilizing strain *Bacillus megaterium* P68 was isolated after being cultured in NBRIP medium for 7 days, and the phosphorus-dissolving capacity could reach 461.86 mg·L^−1^. The application of P68 in field and pot experiments was able to significantly (*p* < 0.05) increase the biomass, total phosphorus content of plants, and tuber yield and improve the AP content of the soil. Furthermore, the transcriptional analysis indicated that strain P68 mainly upregulated processes such as amino acid metabolism, carbohydrate metabolism, and energy metabolism. The growth of potato plants was promoted mainly through glyoxylate and dicarboxylate metabolism, nitrogen metabolism, tryptophan metabolism, alanine, aspartate and glutamate metabolism, pyruvate metabolism, phenylpropanoid biosynthesis, and plant hormone signal transduction. The qRT-PCR analysis of differentially expressed genes showed that inoculated treatments P68 significantly upregulated expression in the phosphate transport, nitrate transport, glutamine synthesis, and abscisic acid regulatory pathways, respectively. The data of qRT-PCR were consistent with that of RNA-seq. In summary, PSB may be involved in the regulation of nitrogen and phosphorus nutrition, glutaminase synthesis, and abscisic acid-related metabolic pathways. This study provided a novel microbial inoculum for potato, and an important data related to the application and production of P68 to improve potato yield. However, the ability of other aspects of P68, such as IAA production, secretion of organic acids, secreting enzymes, potassium solubilization, and colonization ability should be further studied, and additional work should be performed to analyze specific genes involved in the promotion of nutrient uptake in potato when inoculated with PSB.

## Data availability statement

The data presented in the study are deposited in the NCBI repository, accession number PRJNA948577.

## Author contributions

LL: conceptualization, methodology, data curation, software, writing—original draft, and writing—review and editing. CL, ZR, YQ, RW, JW, JC, and LZ: methodology, data curation, formal analysis, and writing—review and editing. XL, XX, and YC: writing—review, editing, project administration, and funding acquisition. All authors contributed to the article and approved the submitted version.

## Funding

This study was supported by the National Natural Science Foundation of China (41977035).

## Conflict of interest

The authors declare that the research was conducted in the absence of any commercial or financial relationships that could be construed as a potential conflict of interest.

## Publisher’s note

All claims expressed in this article are solely those of the authors and do not necessarily represent those of their affiliated organizations, or those of the publisher, the editors and the reviewers. Any product that may be evaluated in this article, or claim that may be made by its manufacturer, is not guaranteed or endorsed by the publisher.

## Footnotes

^1^ https://www.fao.org/faostat/zh/#data/QCL/visualize

^2^ http://ftp.ensemblgenomes.org/pub/plants/release-53/fasta/solanum_tuberosum/
