# Supplementary material for: Transcriptome profiling of genes regulated by phosphate-solubilizing bacteria Bacillus megaterium P68 in potato (Solanum tuberosum L.)
Source: Front Microbiol. 2023 Apr 17;14:1140752. doi: 10.3389/fmicb.2023.1140752 (PMC10150959; doi:10.3389/fmicb.2023.1140752)
Supplement: Supplementary file 1 [file Data_Sheet_1.docx]

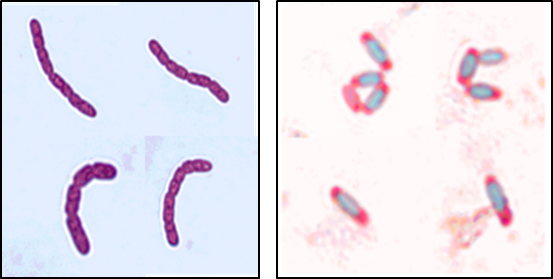


Fig. S1 Microscopy with Gram staining and spore staining of P68. Strain was observed under microscopy as gram-positive, bacteriophage-producing bacteria with rod-shaped bodies


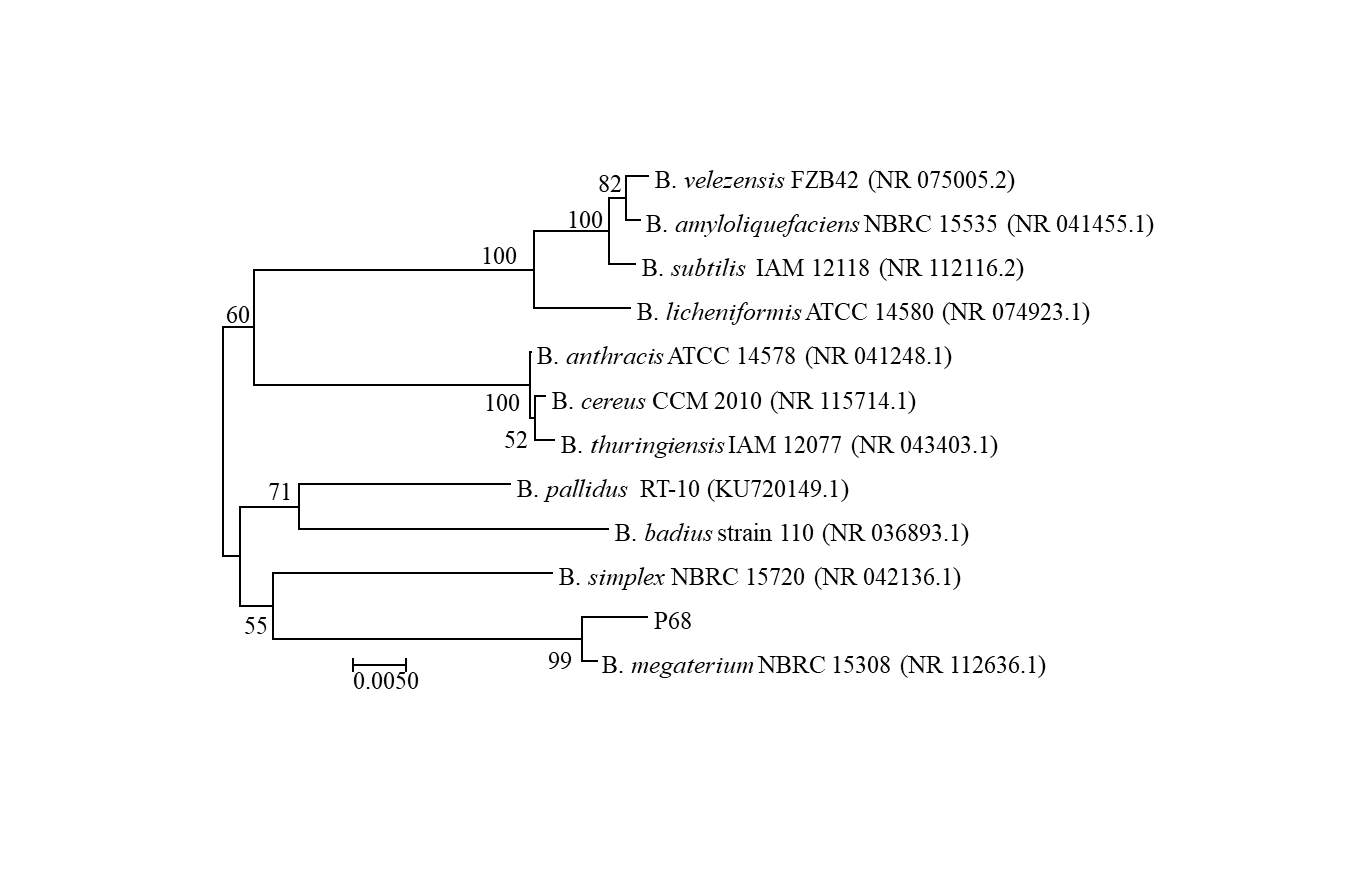


Fig. S2 16S rDNA phylogenetic tree of the strain P68

Table S1 The primers used for real-time PCR (qRT-PCR).

| Selected Genes | Gene name | functional annotations | Forward primer(5’-3’) | Reverse primer(5’-3’) |
| --- | --- | --- | --- | --- |
| Control | EF-1α |  | ATTGGAAACGGATATGCTCCA | TCCTTACCTGAACGCCTGTCA |
| Gene 1 | LOC102592353 | Inorganic phosphate transporter (PGSC0003DMG400010288) | TGAAGATGCCTGAAACGGCT | CCAGTAGCCAGGCACAAATTC |
| Gene 2 | LOC102600280 | Inorganic phosphate transporter (PGSC0003DMG400013451) | CAAGAAAACACGTGGTGCAT | CGAATCCAGCTGATGTGGC |
| Gene 3 | LOC102590802 | Nitrate transporter (PGSC0003DMG400006913) | AGGCCTGTGGGAGGGATAAT | AGCACGCCATAACCAGAACA |
| Gene 4 | LOC125869798 | Glutamine synthetase (PGSC0003DMG400004355) | TGACACTGAGAAGGAAGGCAA | AAGGGTTGGCTCCCACAGTA |
| Gene 5 | LOC102598616 | SAUR family protein (PGSC0003DMG400031744) | GTCCTAGTAGGCGTTTGGGT | GGCGTTTGAACGATCTTGGT |
| Gene 6 | LOC102591866 | Abscisic acid receptor PYL4 (PGSC0003DMG400015897) | TCCCACCAATCACTACAGCG | GGTTGGGTCCCACTAGATGC |


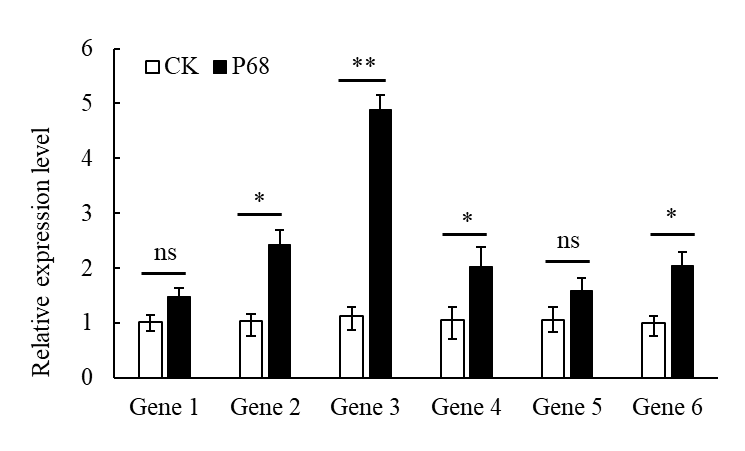


Fig. S3 qRT-PCR relative expression level

The data from qRT-PCR were consistent with those obtained from RNA-seq. The white bar shows relative gene expression level of CK measured by qRT-PCR, and the black bar shows the relative gene expression level of P68. Error bars indicate the standard deviation from three biological replicates. Asterisks represent statistically significant differences (**P < 0.01, *P < 0.05), ns indicates no significant difference as analyzed using Independent-samples’s t-test.
